# Resistance to Water Diffusion in the Stratum Corneum Is Depth-Dependent

**DOI:** 10.1371/journal.pone.0117292

**Published:** 2015-02-11

**Authors:** Mark D. A. van Logtestijn, Elisa Domínguez-Hüttinger, Georgios N. Stamatas, Reiko J. Tanaka

**Affiliations:** 1 Department of Bioengineering, Imperial College London, London, United Kingdom; 2 Johnson & Johnson Santé Beauté France, Issy-les-Moulineaux, France; University Hospital Hamburg-Eppendorf, GERMANY

## Abstract

The stratum corneum (SC) provides a permeability barrier that limits the inflow and outflow of water. The permeability barrier is continuously and dynamically formed, maintained, and degraded along the depth, from the bottom to the top, of the SC. Naturally, its functioning and structure also change dynamically in a depth-dependent manner. While transepidermal water loss is typically used to assess the function of the SC barrier, it fails to provide any information about the dynamic mechanisms that are responsible for the depth-dependent characteristics of the permeability barrier. This paper aims to quantitatively characterize the depth-dependency of the permeability barrier using *in vivo* non-invasive measurement data for understanding the underlying mechanisms for barrier formation, maintenance, and degradation. As a framework to combine existing experimental data, we propose a mathematical model of the SC, consisting of multiple compartments, to explicitly address and investigate the depth-dependency of the SC permeability barrier. Using this mathematical model, we derive a measure of the water permeability barrier, i.e. resistance to water diffusion in the SC, from the measurement data on transepidermal water loss and water concentration profiles measured non-invasively by Raman spectroscopy. The derived resistance profiles effectively characterize the depth-dependency of the permeability barrier, with three distinct regions corresponding to formation, maintenance, and degradation of the barrier. Quantitative characterization of the obtained resistance profiles allows us to compare and evaluate the permeability barrier of skin with different morphology and physiology (infants vs adults, different skin sites, before and after application of oils) and elucidates differences in underlying mechanisms of processing barriers. The resistance profiles were further used to predict the spatial-temporal effects of skin treatments by *in silico* experiments, in terms of spatial-temporal dynamics of percutaneous water penetration.

## Introduction

The main function of the stratum corneum (SC) is to provide a permeability barrier that limits the inflow of external potential irritants, while at the same time controlling the outflow of water from the body to prevent dehydration and retain water [1]. Appropriate hydration in the SC is vital not only for helping to plasticize keratin fibers and provide flexibility to the skin, but also for realization and regulation of all the biochemical processes occurring in the SC by its constituent components [2]. These processes, in turn, contribute to forming, maintaining, and degrading the permeability barrier and to ensuring an appropriate level of SC hydration.

Extensive research has revealed the contribution of many SC components to the permeability barrier. Corneocytes, the main components of the SC, are held together by corneodesmosomes to form tight cellular junctions to provide structural integrity to the SC [3]. They are surrounded by a lipid matrix, where lipid bilayers limit the movement of water and other substances through the SC [4]. Corneocytes contain keratin filaments, filaggrin, and natural moisturising factors (NMFs) that bind water to provide hydration [5]. Each corneocyte is surrounded by an insoluble cornified envelope created by cross-linking of membrane proteins and lipids [6]. Given that all these SC components build up the permeability barrier via different processes that occur dynamically along the depth of the SC, from their formation at the bottom of the SC to their desquamation at the outermost layers, the functioning and structure of the permeability barrier naturally change in a depth-dependent manner.

The permeability barrier is typically assessed through measuring transepidermal water loss (TEWL) [7, 8], which gives a single value that does not, by itself, provide information about the depth-dependency of the permeability barrier. The depth-dependency of the permeability barrier has been previously investigated by measuring TEWL after removal of the SC layers by tape stripping [9–11]. Tape stripping has been also used for depth profiling of several SC components [12]. However, with recent advances in confocal Raman spectroscopy, we are now able to evaluate the depth-dependency of the water permeability barrier using non-invasive *in vivo* measurement data. Accordingly, this paper aims to propose a novel method to combine these non-invasive *in vivo* measurement data and derive the depth-dependent resistance of the SC to water diffusion, using a mathematical model.

While previous pharmacokinetic models of the SC for studying transepidermal drug delivery and absorption mostly assume that the SC is a single compartment with a fixed diffusion coefficient [13, 14], this paper proposes a mathematical model of SC water permeability barrier by assuming that the SC consists of multiple compartments. Each of those compartments is located sequentially at a different depth, and can have a different diffusion coefficient. These novel assumptions enable us to explicitly address and investigate the depth-dependency of the SC permeability barrier. Note that the possibility of representing the SC by multiple compartments has been previously suggested [15, 16] but with the assumption of a constant diffusion coefficient for all the compartments, which does not allow for evaluation of varied permeability along the depth of the SC.

To demonstrate that the proposed mathematical model can effectively evaluate the depth-dependency of the water permeability barrier, we apply the model to previously published experimental data on TEWL and water concentration measured non-invasively at different depths in the SC by confocal Raman spectroscopy. The original studies for these data sets compared the skin of infants and adults [17], different skin sites [18], and the skin before and after application of oils [19]. For each of these data sets, we derive the resistance profiles, which characterise the depth-dependency of the permeability barrier. Quantitative characterisation of the obtained resistance profiles enable us to investigate underlying mechanisms for formation, maintenance, and degradation of the permeability barrier, and their comparison between different skin types can reveal the mechanisms responsible for differences in SC morphology and physiology. Furthermore, our mathematical model allows us to conduct *in silico* experiments to predict the spatial-temporal dynamics of water diffusion in the SC, which is difficult to obtain through conventional experiments. Combining the available TEWL and water concentration data using a mathematical model complements experiments in gaining quantitative understanding of intricate biological mechanisms.

## Results and Discussion

### The SC compartment model

**Fig 1:**
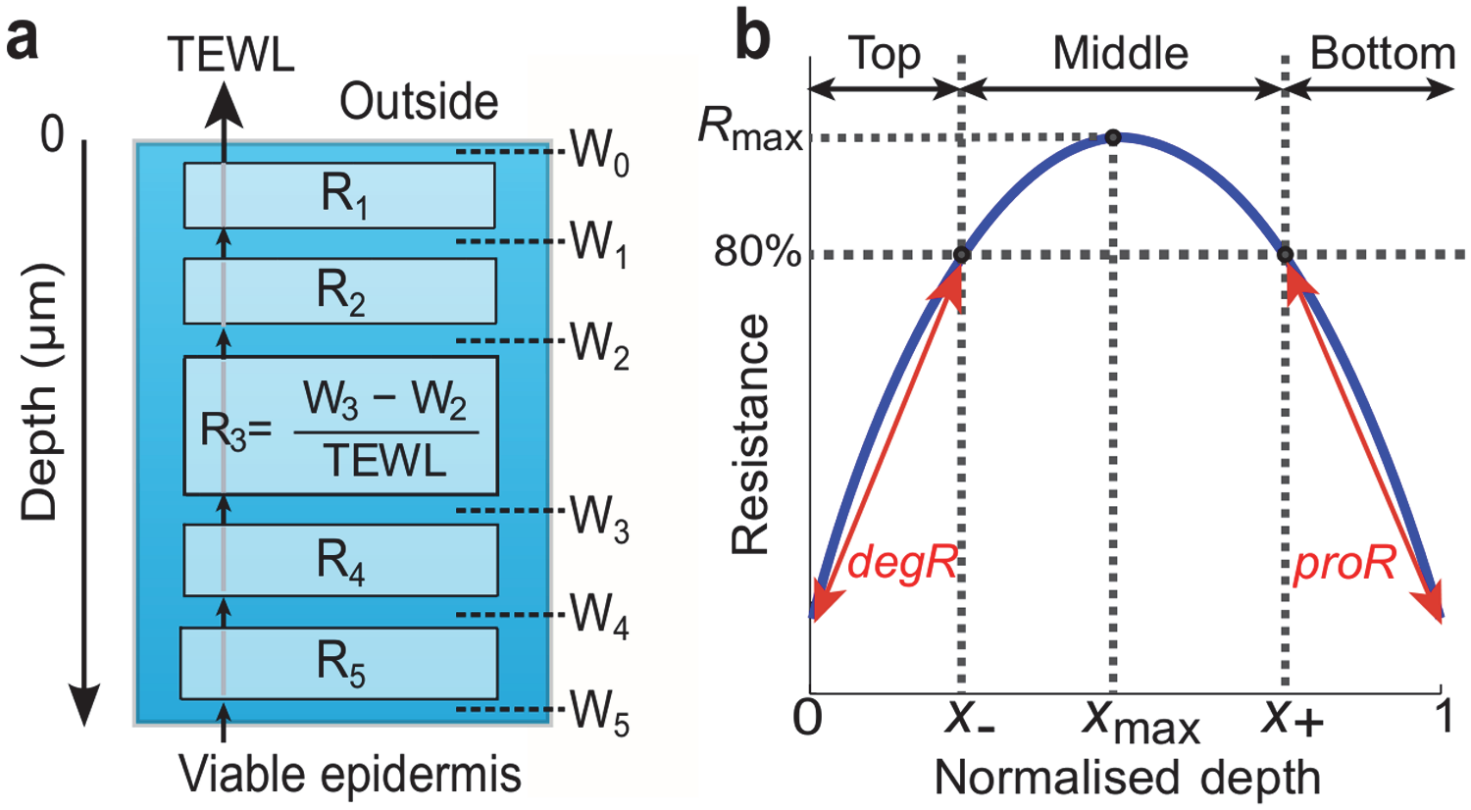
Depth-dependent SC resistance to water diffusion. (**a**) Schematic representation of the SC compartment model (5 compartments). Resistance *R*_*i*_ (*i* = 1,2,…,5) of *i*-th compartment is determined from TEWL and water concentration *W*_*i-1*_ and *W*_*i*_ at the top and bottom of the compartment. (**b**) Typical resistance profile describing SC depth-dependent resistance. Depth is normalized by SC thickness. *R*_*max*_, *degR*, *proR* are functional indices and *X*_-_, *X*_+_ and *X*_max_ are structural indices for resistance profiles.

To study the depth-dependency of the SC water permeability barrier, we develop a mathematical model of the SC consisting of multiple compartments, which we refer to as the *SC compartment model* in this paper. The model incorporates Fick’s first law of diffusion [20] at each compartment at different depths in the SC (Fig. 1a). The application of Fick’s first law determines the relationship between TEWL and the water concentration, measured non-invasively at the top and bottom of the *i*-th compartment (*i* = 1,2,…*n*) of the SC, by

$$\text{TEWL}=D_{i}K_{i}\frac{\Delta W_{i}}{\Delta x_{i}}=\frac{W_{i}-W_{i-1}}{R_{i}},$$ (1)

where *D*_*i*_, *K*_*i*_, Δ*x*_*i*_, and *R*_*i*_ are the diffusion coefficient, partition coefficient of water between the SC and the ambient air, thickness, and resistance of the *i*-th compartment, respectively. The Δ*W*_*i*_ = *W*_*i*_-*W*_*i-1*_ is the difference in water concentration at the top and bottom of the *i*-th compartment, with *W*_*i*_ being the water concentration in steady-state measured at the bottom of the *i*-th compartment. The *W*_*0*_ is the water concentration on top of the outermost compartment, which is in direct contact with the environment, and *W*_*n*_ is the water concentration in the top layer of the viable epidermis. The TEWL for each compartment is the same as TEWL measured conventionally at the top of the SC [21], since the water flux in steady state is equal to the rate of TEWL throughout the SC. The water concentration (% mass ratio water/protein) at sequential depths can be measured by confocal Raman spectroscopy [22].

### Depth-dependent resistance to water diffusion characterizes permeability barrier

Using the SC compartment model (1), we derive the *resistance* of each compartment to water diffusion by $R_{i}=\frac{W_{i}-W_{i-1}}{\text{TEWL}}$, which characterizes how difficult it is for the water to diffuse through the *i*-th compartment. The resistance *R*_*i*_ is also described with both the diffusion coefficient and the partition coefficient as $\frac{\Delta x_{i}}{D_{i}K_{i}}$ from (1), suggesting that both water permeability (diffusion) and binding of water to NMFs (partition) are contributing to the resistance. Plotting the calculated resistances of all the compartments against their respective depth, and interpolating them, provides the *resistance profile* (Fig. 1b). We use the depth normalized by the SC thickness to facilitate the comparison between the resistance profiles of skin with different thickness.

**Fig 2:**
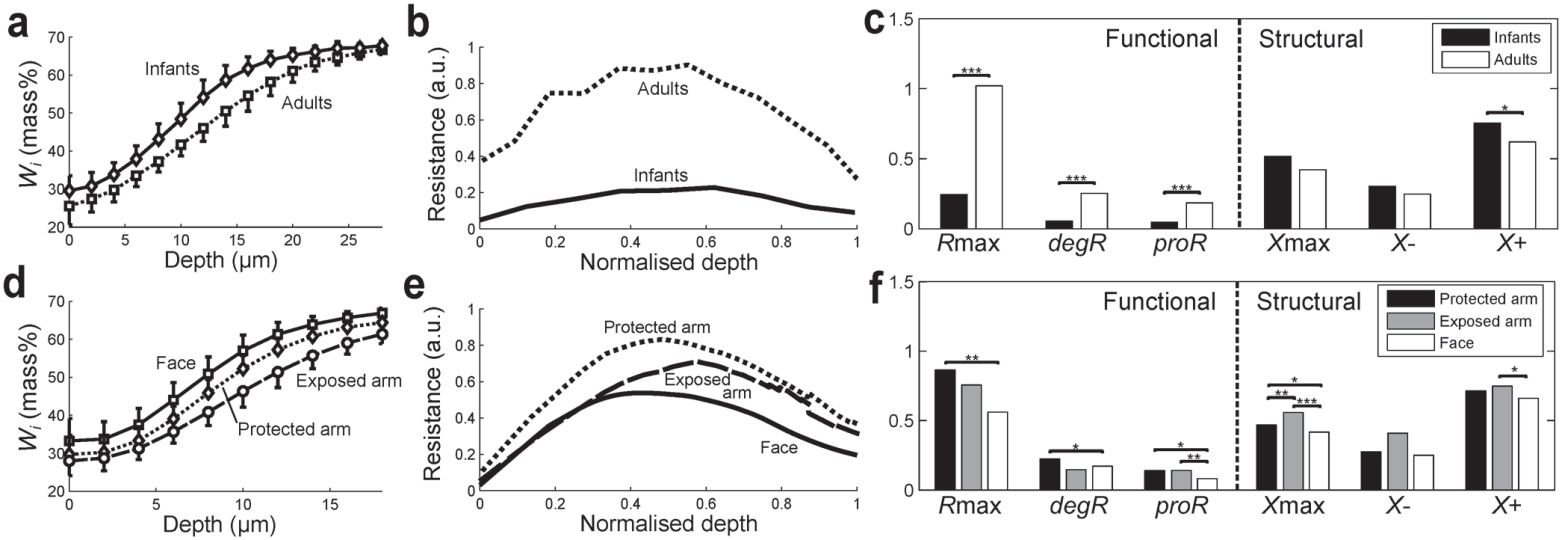
Comparison of permeability barrier for different types of skin. (**a, d**) Water concentration profiles (means +/- SD), (**b, e**) resistance profiles (means), and (**c, f**) quantitative indices for resistance profiles (means, with significance * p<0.05, ** p<0.01, *** p<0.001) for (**a-c**) volar forearm of 12 infants (3–12 months) and 12 adults (14–73 years) and (**d-f**) face (cheek), exposed arm (dorsal forearm), and protected (upper inner) arm of 20 adults (18–70 years).

With regard to the depth-dependency of the permeability barrier, it has been suggested that the SC consists of three distinct regions (bottom, middle, and top) with different “metal-ion barrier properties" [23], with the middle region being the main barrier against diffusion of metal-ions such as potassium and chromium (VI). Despite the difference between metal-ion and water diffusion, we similarly define bottom, middle, and top regions in a typical resistance profile (Fig. 1b), such that each of the regions corresponds to the formation, maintenance, and degradation of the permeability barrier, respectively. The middle region is the area where the resistance is maintained above 80% of the maximum value (*R*_*max*_ attained at depth *X*_max_) throughout. The threshold value of 80% is chosen arbitrarily to capture the distinct region in the middle of the SC, where the profile is relatively flat compared to the other regions and resistance is maintained, as shown in the resistance profile for adults (Fig. 2b). The bottom region comprises the bottom of the SC (normalized depth = 1) to depth *X*+, at which the resistance increases to 80% of *R*_max_. This increase in resistance in the bottom region can be attributed to the processing of various components that constitute the barrier. For example, lipids released from lamellar bodies at the SC/stratum granulosum (SG) interface are processed and self-organized to form the intercellular lipid matrix; the organisation of lipids changes with depth [24]; cross-linking of keratin, membrane proteins, and lipids create an insoluble cornified envelope; tightening of corneodesmosome junctions further strengthens the structure of the SC as a barrier from the SG upwards [25, 26]; corneodesmosome concentration and protease activity also change with depth [27]. The production rate of resistance is defined by $proR=\frac{0.8R_{\text{max}}-R(1)}{1-X+}$, where *R*(1) is the resistance at the SC/SG interface. Finally, the top region is where the resistance decreases from 80% of *R*_max_ at depth *X-* towards the outermost layer (depth = 0). This decrease in resistance is due to active kallikreins (desquamatory serine proteases) degrading corneodesmosomes and the resulting removal of corneocytes and lipids [28]. The degradation rate of resistance is defined by $degR=\frac{0.8R_{\text{max}}-R(0)}{X-}$, where *R*(0) is the resistance at the outermost compartment.

The resistance profiles are quantitatively characterized by *R*_max_, *proR*, and *degR*, all of which indicate the functioning of the SC as a water permeability barrier and are referred to as functional indices. The term *R*_max_ indicates the overall strength of the barrier resistance, higher *proR* values imply more efficient mechanisms of barrier construction, and *degR* indicates the efficiency of desquamation processes to degrade the barrier towards the uppermost layer. The spatial characteristics of the resistance profiles can be quantified by *X*_max_, *X*-, and *X*+, which we refer to as structural indices. The term *X*_max_ specifies where the resistance peaks in the SC, higher *X*+ values indicate that the barrier is formed within a smaller region of depth, and higher *X*- values suggest that the barrier degradation starts deeper in the SC. Both functional and structural indices enable us to quantitatively compare the resistance profiles obtained for different skin types and help us to study the contributions of constituent barrier components to formation, maintenance and degradation of the permeability barrier, as discussed below.

### Age- and site-dependent differences in permeability barrier captured using resistance profiles

To exemplify how the resistance profiles provide insights in the underlying mechanisms related to the depth-dependent permeability barrier, we first apply the SC compartment model to previously published data on water concentration profiles (Fig. 2a) and TEWL [17] to derive the resistance profiles for adults and infants of 3–12 months old (details in Methods). Infant skin undergoes a maturation process during the early years of life, starting as early as 3 months old [17, 29], when the morphology and physiology of the SC barrier fully develops [30]. The immaturity of the SC barrier for infants of this age (3–12 months old) is conventionally documented by higher TEWL values compared to adult skin [17, 29], although TEWL for babies immediately after birth is reported to be almost the same as that for adults [31]. The resistance profiles for adults and infants (Fig. 2b) further reveal the depth-dependent function and structure of the SC as a water permeability barrier. The resistance profile for infants clearly shows a much lower resistance than for adults throughout the thickness of the SC. This lower resistance in infants is reflected in significantly lower values for all the functional indices (*R*_max_, *proR*, and *degR*, Fig. 2c), indicating a less efficient barrier function compared to adult skin. In particular, a lower *proR* for infants could be attributed to their higher keratinocyte turnover rates due to increased proliferation [32]. This means that cells move up quicker and have shorter resident times in the epidermis, resulting in less mature differentiation and cornification and thus a less efficient formation of the barrier. Interestingly, however, the differences observed in structural indices (*X*_max_, *X*- and *X*+) are not as significant, suggesting that the processes related to formation, maintenance and degradation of the permeability barrier are activated at similar relative depths in the SC.

We also derived and compared the resistance profiles for different skin sites on adults using previously published data on water concentration profiles (Fig. 2d) and TEWL [18]. It is well known that the face has a thinner SC [33] and higher TEWL values [7] than the arms, and they are conventionally used as an indication of a weaker permeability barrier for the face. The weaker resistance for the face can indeed be observed clearly in our calculated resistance profiles (Fig. 2e) and is quantitatively indicated by lower values for all the functional indices (Fig. 2f). The lower *proR* for the face reflects a less efficient formation of the barrier, possibly due to higher rates of proliferation [34], as in the case for infants. This also results in shifting the peak of the resistance towards the top of the SC (lower *X*_max_) with a lower *R*_max_. Comparison of the resistance profiles between protected and exposed arms reveals the effects of photo-ageing caused by chronic exposure to UV radiation, which damages the epidermis and impairs its differentiation processes [35, 36]. The exposed arm has a lower peak in resistance (lower *R*_max_) than the protected arm, achieved at a deeper point (larger *X*_max_), possibly due to impaired differentiation processes leading to lower availability of barrier component precursors, which limit the barrier formation.

### Effects of topical application of oil evaluated using resistance profiles

We further use the resistance profiles to study and predict the effects of skin treatments on the permeability barrier. Pharmaceutical skin care products, such as oil-based moisturizers and emollients, aim to improve SC barrier function by partially occluding the skin, limiting the outflow of water and consequently trapping water in the SC to increase its hydration levels [19]. To quantitatively evaluate and compare the effects of topical application of different oils in increasing the resistance to water diffusion, we applied the SC compartment model to the previously published data on TEWL and water concentration profiles [19, 37]. It allowed us to derive the resistance profiles for the skin before and 30 minutes after topical application of four oils: almond, jojoba, paraffin, and petrolatum. The measurements at 30 minutes after application of the oils are considered to be at steady state [19].

**Fig 3:**
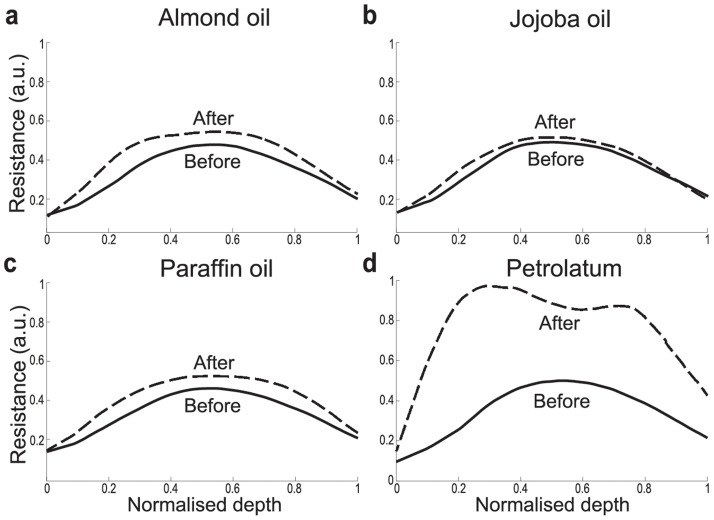
Resistance profiles (means) before and 30 minutes after topical application of different oils. (**a**) almond oil (*n* = 92), (**b**) jojoba oil (*n* = 98), (**c**) paraffin oil (*n* = 99), and (**d**) petrolatum (*n* = 86).

Topical application of all oils increased the resistance over the entire depth of the SC, in a depth-dependent manner, with a higher increase observed in the middle to top than the bottom regions (Fig. 3). This suggests that the permeability barrier is not only affected by oils through occlusion of the skin since the subsequent decrease in TEWL would simply scale up the resistance profiles in a depth-independent manner. The depth-dependent increase in resistance can be partly explained by local increase in hydration. The increase is highest in the middle region of the SC [38], where most of the NMFs reside and bind to water to prevent its diffusion, resulting in increased resistance against diffusion of water. This result is also consistent with the findings that the middle region is the main barrier against diffusion of metal-ions [23].

Application of petrolatum almost doubles the resistance on average over the entire SC, while the maximum increase in the resistance by application of the other three oils is about 20% (Fig. 3d). This strong increase in resistance by application of petrolatum could be due to a larger increase in hydration caused by a larger occlusive effect of petrolatum and a higher concentration of petrolatum reaching deeper in the skin than the other oils [19], to strengthen the intercellular lipid bilayers and increase the resistance across the SC. Interestingly, two peaks are observed in the resistant profile after the topical application of petrolatum. This could be caused by molecular fractionation due to variation in speed of lipid transfer within the SC, which resembles the processes of liquid chromatography [39].

### *In silico* simulation reveals the effects of topical application of petrolatum on spatial-temporal dynamics of water diffusion

To analyze the effects of topical application of petrolatum on the spatial-temporal diffusion of water in more detail, we developed a mathematical model, which describes the dynamical water inflow and outflow of each compartment, using the resistance profiles obtained above.

The dynamical change in the water concentration *W*_*i*_ in the *i*-th compartment can be described by an ordinary differential equation (ODE),

$$\frac{dW_{i}(t)}{dt}=\frac{W_{i-1}(t)-W_{i}(t)}{R_{i}}+\frac{W_{i+1}(t)-W_{i}(t)}{R_{i+1}},$$ (2)

where *R*_*i*_ and *R*_*i+1*_ are resistances limiting the diffusion of water to and from the *i*-th compartment. The set of ODEs (2) for *i* = 1,…,*n*-1 is referred to as the SC compartment *dynamic* model, which describes the spatial-temporal dynamics of water diffusion. Using the SC compartment dynamic model, we carried out *in silico* simulations of SC water absorption and desorption (details in Methods). These simulations mimic the conditions used in previously published experiments [17], in which water is applied topically to the skin for 10 seconds before being wiped off.

**Fig 4:**
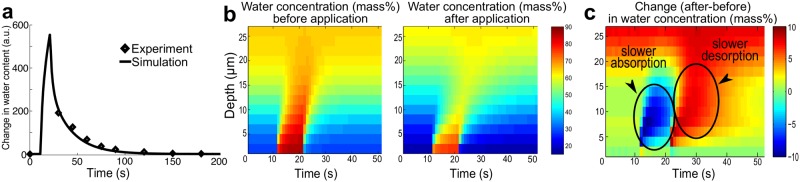
Results of *in silico* absorption-desorption experiment with water applied topically for 10 seconds (time = 10–20 s). (**a**) Temporal dynamics of total water content (change from steady state) for adults; simulation results (solid line) and experimental data (diamonds) [17]. (**b**) Spatial-temporal dynamics of water concentration before and 30 minutes after topical application of petrolatum. (**c**) Spatial-temporal increase in water concentration after application of petrolatum. Negative values (time = 10–20 s) and positive values (time = 20–50 s) respectively indicate slower absorption and desorption after application of petrolatum.

Whereas the original experiments [17] involved measuring the total SC water content at different time points and derived the rates of absorption and desorption of water to quantify the barrier function, our *in silico* experiments further predicted the temporal dynamics of the spatial diffusion of water in the SC. Assuming that the total water content is obtained by summing up the water concentrations in all the compartments along the depth, we compared and confirmed the qualitative match between the total SC water content measured during the experiments for adults [17] and that obtained from our simulations for the skin of adults before topical application of petrolatum (Fig. 4a).

Our simulation can visualize the effectiveness of topical application of petrolatum in improving the permeability barrier in terms of spatial-temporal water diffusion, which is difficult to observe through conventional experiments. Since petrolatum is generally applied to protect skin with a damaged barrier [40], e.g., for atopic dermatitis patients, detailed understanding of the dynamic effects of petrolatum and other emollients is clinically relevant. The simulation results clearly indicate that the area with high water concentration shrinks by topical application of petrolatum (Fig. 4b). This is due to the change in kinetics, as the increased resistance by petrolatum prevents water from flowing in and out. It is exhibited by the spatial-temporal change in water concentration by petrolatum application (Fig. 4c): a slower absorption of water penetrating less deep for the first 10 seconds while water is applied, and a slower desorption at around 25–35 seconds leading to the increased water concentration to be sustained.

## Conclusions

The critical function of the skin acting as a barrier to water diffusion and to tissue dehydration is typically assessed by measuring TEWL. The TEWL however is a single value that provides no information about the dynamic mechanisms (e.g. barrier formation or degradation) that are responsible for the depth-dependent characteristics of the permeability barrier. This paper proposed the SC compartment model, used it to combine existing static TEWL data and water concentration profiles, both of which can be measured non-invasively *in vivo*, and extracted information on depth-dependency of the permeability barrier. Our method used multiple compartments with depth-dependent diffusion coefficients in the proposed SC compartment model, combined with *in vivo* non-invasive measurements. This proposed method is a refinement of previously published one with single compartment SC models combined with tape stripping experiments [9, 10] (detailed comparison in S1 Information). We used the derived resistance profiles to evaluate the permeability barrier of different skin types, and elucidated differences in the underlying mechanisms of barrier formation, maintenance and degradation.

The obtained resistance profiles revealed a depth-dependent water permeability barrier, which peaks at the middle region of the SC, indicating that the barrier is formed, maintained, and degraded from the bottom to the top of the SC. Quantitative characterization of the resistance profiles allowed us to compare the permeability barrier of different skin types (infants vs. adults, different skin sites, before and after application of oils) and lead to the following observations on the underlying mechanisms. Higher proliferation rates both in infants (compared to adults) and in the face (compared to arm sites) lead to a less efficient formation of the barrier, indicated by a slow formation of the barrier (low *proR*) with lower maximum resistance (low *R*_max_). Photo-ageing by UV radiation impairs differentiation processes and limits barrier formation, as exhibited by a lower peak in resistance (low *R*_max_) achieved at a deeper point in the SC (high *X*_max_) on the exposed arms (compared to the protected arms). The increased resistance after topical application of petrolatum leads to less penetration of water and a prolonged increase in water concentration, as predicted by *in silico* experiments for spatial-temporal dynamic of water diffusion in the SC.

Our mathematical modelling approach provides a tool for the analysis of experimental data for a deeper understanding of the mechanisms underlying barrier function. It can be used to predict the effects of skin treatments on spatial-temporal dynamics of water penetration in the SC, through *in silico* experiments. This exemplifies the power of appropriate mathematical frameworks providing additional insights in underlying dynamical mechanisms, which cannot be obtained solely through experiments [41]. This approach can also be applied to obtain clinical insights, for example by quantifying the effects of skin treatments in improving barrier function, using resistance profiles for different skin types and sites with pathological skin barrier conditions (such as atopic dermatitis and psoriasis) or after topical application of various drugs and skin care products such as corticosteroids or moisturizers. Similar *in silico* simulations to those presented in this paper can be used to study the spatial-temporal dynamics of percutaneous penetration of drugs and absorption kinetics of allergenic substances and may provide insights into the key biological processes involved in disease states or in treatments and identify patient-specific SC barrier dysfunction and targeted treatments.

Non-invasive measurement techniques allow for accurate measurement of depth-dependent concentrations of NMF, lipids, and proteins [42, 43], in addition to water concentration used in this paper. As such detailed quantitative measurements for SC components become increasingly available, appropriate mathematical frameworks for combining multiple sets of experimental data, such as the one proposed here, are needed to maximize the insights that can be gained from measurement data, and progress our understanding of the SC permeability barrier. While the current model serves as a first step for modeling permeability in the skin, its flexibility and simplicity will allow us to extend the model to incorporate other spatial-temporal processes, such as water binding to NMFs, and to reveal further how various biological mechanisms contribute to barrier formation, maintenance, and degradation, for example by clarifying the relative contribution of both water binding to NMF (hydration) and the extracellular lipids to resistance to water diffusion.

## Methods

### Derivation of resistance profiles

The resistance profiles describing the depth-dependent resistance to water diffusion (Figs. 2 and 3) are obtained in the following three steps, using MATLAB version 2012b (The MathWorks, Inc., MA, USA). First, resistances $R_{i}=\frac{W_{i}-W_{i-1}}{\text{TEWL}}$ are calculated with the SC compartment model (1) using TEWL [*g|h|m* ^2^] and water concentration [mass%] measured by Raman spectroscopy for each individual [17–19] (all the data used in our paper is summarised in S1 Dataset). The thickness of each compartment in the compartment model is set to be 2 *µ*m, corresponding to the measurement of water concentration at every 2 *µ*m in depth. The unit of resistance using this specific combination of measurements is displayed as arbitrary units (a.u.), since [mass%] is a relative unit (% mg water/mg protein). Second, the resistance profile for each individual is obtained by plotting the resistances against the normalized depth (depth divided by the SC thickness) and interpolating them. The SC thickness is calculated from the water concentration profiles by the method described in [44]. The Matlab code to derive the resistance profiles for each individual is available in (S1 Code). Finally, all the resistance profiles are averaged over the group of individuals to obtain the mean resistance profile for the corresponding group. The functional and structural indices are also calculated for every individual resistance profile and averaged over the group (Fig. 2).

### *In silico* experiments

The *in silico* absorption-desorption experiments (Fig. 4) were performed using simulation of the SC compartment dynamic model (Equation (2)). The resistances {*R*_*i*_} are obtained from the resistance profiles, and the water profiles are obtained from the previously published measurement data [17, 19]. Topical application of water is simulated by increasing *W*_0_ by *W*_exp_ = 60 [mass%] for 10 seconds. The value of *W*_exp_ was chosen arbitrarily to ensure that the pool of water applied on top of the skin diffuses from the top to deeper SC layers due to the difference in water concentration, with *W*_0_ being around 90 [mass%] after the application of water, while a typical initial *W*_0_ is 30 [mass%]. Numerical simulation were conducted using the *ode45* solver of MATLAB version 2012b (The MathWorks, Inc., MA, USA). The Matlab code can be found in S1 Code.

Direct comparison between the experimental and model simulation results was carried out as follows. We first ran the simulation using the SC compartment dynamic model (2) to obtain the spatial-temporal dynamics of water concentration before the application of petrolatum. Summation of the water concentration in all the compartments along the depth provided the simulated time-course of total SC water content [mass%]. To directly compare it to the total SC water content [a.u.] experimentally measured by conductance, we obtained a scaling parameter for the unit conversion by minimizing the sum of squared errors between the simulation and experimental data at each time point using *fminunc* in Matlab. We confirmed that the results are robust against the choice of initial values for minimization.

### Statistical analysis

The statistical significance of the difference between the means of indices was determined using a two-tailed Student’s t-test for comparing the means of infants and adults and using one-way ANOVA for comparing the means of the three skin sites (MATLAB version 2012b, The MathWorks, Inc., MA, USA). The variance in the mean resistance profiles is not displayed (Fig. 2b, e) for visual clarity. However, quantitative indices were used to determine the statistical significance between the resistance profiles and their characteristics; thus, they include variance.

## Supporting Information

S1 Dataset Experimental data on water concentration and TEWL. This dataset contains the experimental data used in this paper. It includes water concentration profile and TEWL data on infants and adults [17], different skin sites [18], and before and after application of four oils [19].
(XLSX)

S1 Information Comparison of results on depth-dependency to previously published results using tape stripping experiments. (PDF)

S1 Code Matlab code for reproducing the results in this paper and in the S1 Information. (M)
