# Supplementary material for: Resistance to Water Diffusion in the Stratum Corneum Is Depth-Dependent
Source: PLoS One. 2015 Feb 11;10(2):e0117292. doi: 10.1371/journal.pone.0117292 (PMC4324936; doi:10.1371/journal.pone.0117292)
Supplement: S1 Information — (PDF) [file pone.0117292.s002.pdf]

Resistance to water diffusion in the stratum corneum is depth-dependent

Mark D. A. van Logtestijn, Elisa Domínguez-Hüttinger, Georgios N. Stamatas and Reiko J. Tanaka

Supporting Information S1

Comparison of results on depth-dependency to previously published results using tape stripping experiments

Our paper proposes a SC compartment model in order to investigate the depth-dependent characteristics of the SC permeability barrier. The proposed SC compartment model, together with non-invasive *in vivo* measurements on TEWL and water concentration, clarifies the depth-dependent 'three-layer' structure of the SC permeability barrier.

Previously, the depth-dependency of the SC permeability barrier has been studied mainly by tape stripping, in combination with a single compartment model [9,10]. By applying Fick's first law of diffusion to the SC, and considering the SC as one compartment, they have claimed that a linear fit of the plot for "1/TEWL vs SC thickness removed" (1/TEWL plot) implies that the diffusion coefficient is independent of the SC depth, concluding that the SC permeability barrier is "homogeneous" across its depth.

This supplementary file clarifies where the different conclusions on the depth-dependency of the SC permeability barrier (depth-dependent or "homogeneous") come from. We specifically demonstrate that

1) Data for depth-dependent permeability barrier [17, 18] can reproduce the "homogeneity" in the 1/TEWL plot,
2) Depth-dependent resistance profiles can be obtained from data for "homogeneous" barrier [9, 10].

1) Data for depth-dependent permeability barrier can reproduce the "homogeneity" in the 1/TEWL plot

To demonstrate that the data for depth-dependent SC barrier [17, 18] can reproduce the "homogeneity" as in [9,10], we conduct *in silico* tape-stripping experiments using our SC compartment model. The "homogeneity" is evaluated by calculating TEWL after tape stripping of each layer.

In our SC compartment model, TEWL is described by $TEWL = \frac{\sum_{i=1}^{n} \Delta W_i}{\sum_{i=1}^{n} R_i} = \frac{W_n - W_0}{\sum_{i=1}^{n} R_i}$. It is obtained by summing up the resistance *Ri* of the *i*-th compartment $R_i = \frac{\Delta W_i}{TEWL}$ (Equation (1)) for all the compartments (i=1,…,n) as $\sum_{i=1}^{n} R_i = \frac{\sum_{i=1}^{n} \Delta W_i}{TEWL}$. This representation of TEWL suggests that the water flow is driven by the difference in water concentration between the viable epidermis (W_n) and environment (W_0), since $W_n - W_0 = \sum_{i=1}^{n} \Delta W_i$. The TEWL after tape stripping of the top x compartments is calculated by $TEWL_x = \frac{W_n - W_0}{\sum_{i=x+1}^{n} R_i}$, by setting the resistance of all the removed compartments to be zero. The removed compartments do not contribute to the total resistance that water encounters when it flows from the viable epidermis to the outside environment. These *in silico* tape stripping experiments thus provide TEWLx for different depth x, or SC thickness removed, using the resistance profiles {*Ri*} and the water concentration at the top and bottom layers (W_0 and Wn).

Our paper demonstrated the depth-dependent resistance profiles for skin at different sites and for different ages using the TEWL and water profile data in [17, 18]. Using each of the depth-dependent resistance profiles, we derived TEWLx by *in silico* tape stripping experiments described above (Matlab code can be found in Code S1). The resulting 1/TEWL plots (e.g. Figure S1) exhibit a similar linear decrease in

1/TEWLx against the SC thickness removed (x), as in 1/TEWL plots obtained from the actual tape stripping experiments (e.g. Figure 7 in [9]). This agreement confirms the validity of the *in silico* tape stripping experiments, using the SC compartment model, to directly compare the results from *in silico* and actual tape stripping experiments.

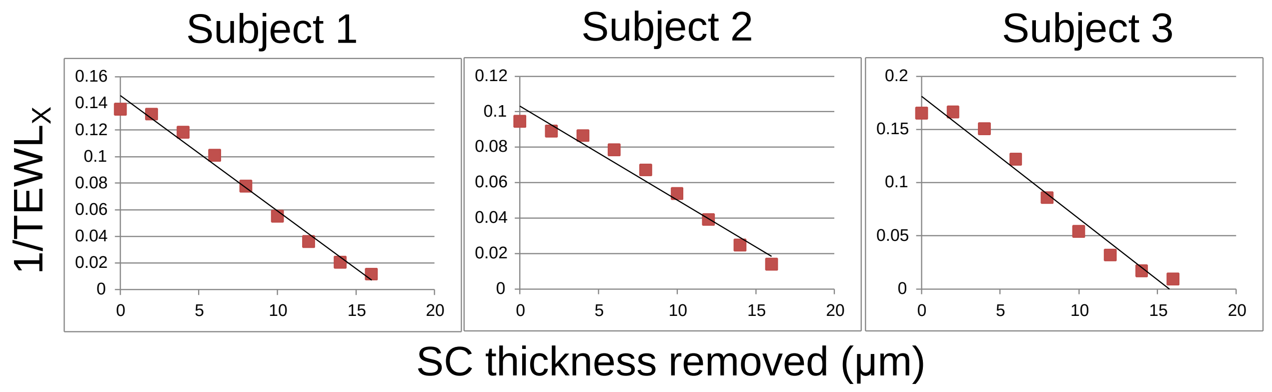

Figure S1 1/TEWL plots obtained by *in silico* experiments using data for three randomly chosen subjects for adult arm [18].

Linear fitting of the 1/TEWL plots obtained by *in silico* tape stripping experiments using the data in [17, 18] (e.g. Figure S1) results in high r^2 values, in the range of 0.94 ~ 0.995 (e.g. Figure S2), which is in a similar range as those obtained by actual tape stripping experiments [9,10]. These high r^2 values suggest that data for depth-dependent SC barrier (with depth-dependent resistance profiles, e.g. Figure 2(e) in the manuscript) exhibit the "homogeneity" as claimed previously in [9,10], where homogeneity means that the corresponding 1/TEWL plot is linear. Therefore, the linearity of the 1/TEWL plot is not sufficient to claim that the permeability barrier is depth-independent.

This paradoxical conclusion of the homogeneity for a non-homogeneous barrier results from the implicit assumption made when the 1/TEWL plots are fitted to a linear line. Linear fitting of the 1/TEWL plots implicitly assumes the "homogeneity" with respect to the SC thickness removed, by assuming that the SC is represented as a single compartment, with a unique and depth-independent diffusion coefficient D in Fick's first law of diffusion. The linear fitting, under the assumption of a single D, can therefore inevitably lead to the conclusion of the homogeneity even for a non-homogeneous barrier.

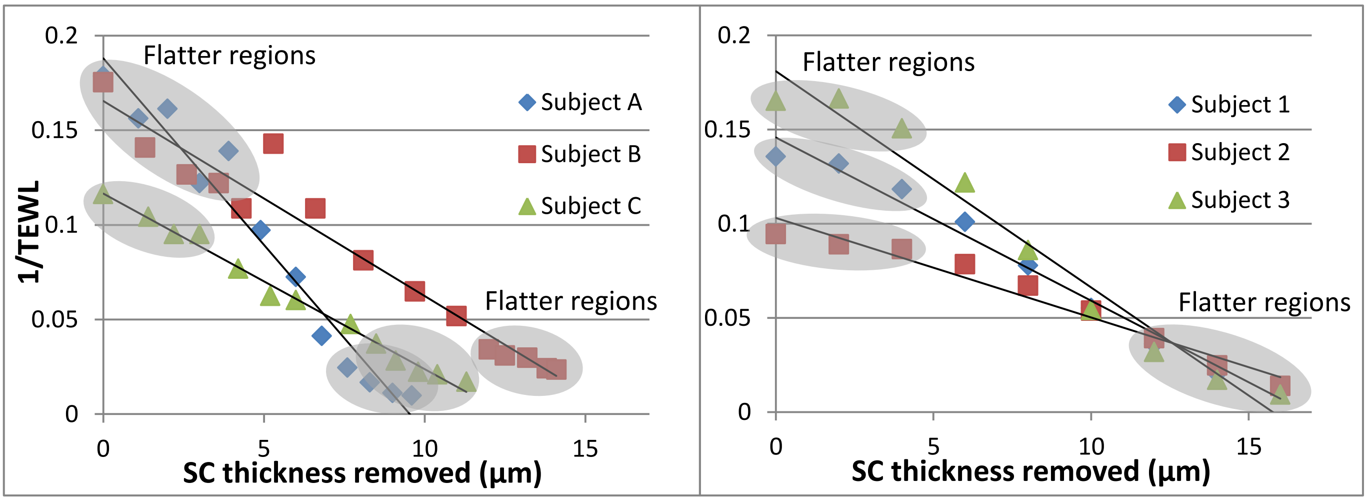

Figure S2 Linear fitting of the 1/TEWL plots obtained by (a) actual experiments (data from Table 1 in [9]) and (b) *in silico* experiments.

A closer look at the 1/TEWL plots in Figure S2 clarifies that the linear slope actually flattens for a few μm at the top and bottom of the SC (shown as "flatter regions" in Figure S2), that is dismissed in a linear fit of the data under the assumption of a single compartment SC model. This flattening of the data at the bottom and top SC layers suggests the possible depth-dependent characteristics of the SC, as they are caused by increase in the resistance at the bottom of the SC (*proR*) and the decrease in resistance at the top (*degR*). Although the linear fit gives a first approximation of the 1/TEWL plots, our SC compartment model makes the most of the depth-dependent non-invasive *in vivo* measurement data (water concentration profile and depth) to study the depth-dependent characteristics of the SC resistance in more detail, as the model consists of multiple compartments with possibly different diffusion coefficients.

2) Depth-dependent resistance profiles can be obtained from data for "homogeneous" barrier

In silico tape stripping experiments described above derive 1/TEWL plots from resistance profiles. A similar method, in the reverse order, can derive resistance profiles from 1/TEWL plots obtained by tape stripping experiments. The resistance R_i for the *i*-th compartment is obtained by $R_i=(W_n-W_0)(\frac{1}{TEWL_{i-1}}-\frac{1}{TEWL_i})$, since $\sum_{i=x+1}^{n} R_i=\frac{W_n-W_0}{TEWLx}$ from $TEWL_x=\frac{W_n-W_0}{\sum_{i=x+1}^{n} R_i}$.

We applied this method to the data (TEWL and corresponding depth) obtained by tape stripping experiments (Subject A in Kalia et al. [9], Table 1). The Matlab code is available as supporting information (Code S1). The derived resistance profile (Figure S3) exhibits a good fit with a parabola (blue line in Figure S3), a shape similar to a typical resistance profile. If the resistance is depth-independent (homogeneous), the resistance profiles would fit to a horizontal line. This result confirms that the SC compartment model allows us to derive resistance profiles, using the data from tape stripping experiments, to reveal the depth-dependency of SC permeability barrier.

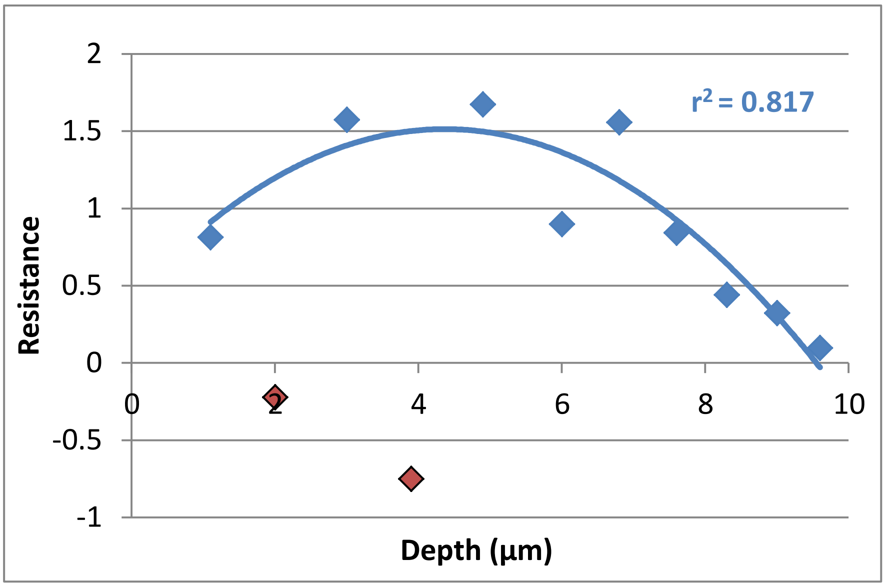

Figure S3 The resistance profile derived from the data from tape stripping experiments (Subject A in Kalia et al. [9], Table 1), with a parabolic fit (r^2=0.817). Two data points (shown in red), exhibiting decrease in TEWL after tape stripping (negative resistance values) are not included in the fitting.

Conclusion

We have shown that both confocal Raman spectroscopy and tape stripping experiments can be equally used to study the depth-dependency of the SC water permeability barrier, using our proposed SC compartment model. Resistance profiles can be obtained using data from tape stripping experiments and allow us to conduct *in silico* tape stripping experiments.

These methods reveal the depth-dependency of the SC resistance to water diffusion, with the resistance decreasing at both the top and bottom ends of the SC. The key assumption in our SC compartment model is the multiple compartments with possibly different diffusion coefficients to represent the SC. A linear fitting to 1/TEWL plots obtained by tape stripping experiments assumes the SC to be one compartment with a fixed diffusion coefficient, and thus dismisses the depth-dependency. We have demonstrated an example that interpretation of 1/TEWL plots obtained by tape stripping experiments, using the SC compartment model with multiple compartments, revealed a depth-dependent ‘three layer’ resistance profile.

Depth-dependent resistance of the SC is a natural consequence of the fact that the SC components, which constitute the resistance, are built and degraded in a depth-dependent manner, as opposed to a constant resistance of the barrier which is built and degraded abruptly. For example, the resistance of the SC top layer decreases due to the desquamation. It is in concordance with data obtained by tape stripping experiments, e.g. Figure 5 in Kalia *et al*. [9] demonstrating that TEWL hardly increases after removal of the first 4 µm.
